# Information Sources, Risk Perception, and Efficacy Appraisal’s Prediction of Engagement in Protective Behaviors Against COVID-19 in China: Repeated Cross-sectional Survey

**DOI:** 10.2196/23232

**Published:** 2021-01-12

**Authors:** Jian Raymond Rui, Keqing Yang, Juan Chen

**Affiliations:** 1 College of Journalism and Communication South China University of Technology Guangzhou China

**Keywords:** information source, perceived severity, perceived susceptibility, response efficacy, self-efficacy, health information, protective behavior, COVID-19, protection, behavior, risk, perception, prediction

## Abstract

**Background:**

As the COVID-19 pandemic has become a major public health threat worldwide, it is critical to understand what factors affect individual engagement in protective actions. Because of its authoritarian political system and state-owned media system, how Chinese individuals engaged in protective actions against COVID-19 might be different compared to other countries.

**Objective:**

The purpose of this study is to examine how the source of information about COVID-19, Chinese individuals’ risk perception of COVID-19 (ie, perceived severity and perceived susceptibility), and their efficacy appraisal in controlling COVID-19 (ie, response efficacy and self-efficacy) affected their engagement in protective actions. Additionally, this study aims to investigate whether there is any difference in these relationships throughout the duration of this pandemic.

**Methods:**

A six-wave repeated cross-sectional survey (N=1942) was conducted in six major cities in China between February 7 and April 23, 2020. Participants’ reliance on expert versus inexpert sources for information about COVID-19, their perceived severity of and susceptibility to COVID-19, their response efficacy and self-efficacy, and their engagement in protective actions (staying at home, wearing a face mask, and washing hands) were measured. Demographic variables (sex, age, income, education, and city of residence), knowledge of COVID-19, and self-rated health condition were controlled.

**Results:**

Reliance on expert sources did not become the major factor that motivated these actions until wave 3, and the negative effect of inexpert sources on these actions was limited to wave 2. Perceived severity encouraged some protective behaviors but its effect varied depending on the specific behavior. In addition, perceived severity exhibited a stronger effect on these behaviors compared to perceived susceptibility. The positive effect of response efficacy was only significant at waves 1 and 2, and limited to certain behaviors.

**Conclusions:**

Chinese individuals’ engagement in protective behaviors might not entirely be their autonomous decision but a result of compliance with executive orders. After the early outbreak, expert sources started to facilitate protective behaviors, suggesting that it might take time to develop trust in these sources. The facilitating effect of perceived severity lasted throughout the duration of the pandemic, but that of response efficacy was limited to the early stage.

## Introduction

### Background

Having spread to 188 countries and regions [1], COVID-19 has become a serious public health threat worldwide. As of October 16, 2020, COVID-19 has caused over 38 million cases and nearly 1,100,000 deaths [1]. China is the first country where COVID-19 was discovered. As early as December 2019, COVID-19 was found in Wuhan, China [2]. The number of confirmed cases and deaths in China grew rapidly in January but started to decline in late February [3]. As of October 16, 2020, China reported 90,899 cases including 4739 deaths [1].

Despite scientific efforts, much about COVID-19 still remains uncertain, such as its origin and mutation [4]. Thus, given its high levels of risks, individuals are encouraged to take protective actions [5,6]. The extant research has explicated how individuals’ engagement in protective behavior against COVID-19 varied depending on their knowledge, fear, risk perception, morality, and internet use [7-12].

However, empirical evidence in China is still scarce. The authoritarian political system in China reduced resistance to the government’s executive orders such as locking down cities and placing citizens under quarantine [13], which controlled the spread of the pandemic [3]. In addition, the state ownership of media in China enables the government to provide large-scale health education and campaigns consistently, which might have facilitated engagement in protective actions. Thus, investigations on what factors affected Chinese individuals’ engagement in protective actions against COVID-19 may provide additional knowledge on the potential influence of a unique sociocultural environment on health behavior.

However, to the best of our knowledge, only one study was conducted on how Chinese individuals performed protective actions against COVID-19 [12]. Furthermore, that study is a one-time cross-sectional investigation at the early stage of the outbreak [12]. As it remains unknown when the pandemic might end, it is critical to examine how factors related to taking preventive measures against COVID-19 might change across different stages of its outbreak. Therefore, this study employs a repeated cross-sectional approach to address this limitation. Specifically, built on the extended parallel process model (EPPM) [13], this study aims to test the theory by examining how individuals’ risk perception of COVID-19 and their efficacy appraisal in controlling COVID-19 might affect their engagement in protective actions. Moreover, we seek to add to the extant research by investigating the role that one’s reliance on different sources for information about COVID-19 plays in performing these protective actions. Although we built our study on EPPM, other theoretical work such as the protection motivation theory [14], the health belief model [15], and the risk perception attitude framework [16] also considered variables in EPPM and made similar predictions.

### EPPM

EPPM contends that whether individuals engage in protective behaviors depends on their risk perception and efficacy appraisal [17]. Risk perception is usually conceptualized as the sum or average of perceived severity and perceived susceptibility [18]. Perceived severity refers to one’s perception of the adversity of consequences if individuals do not engage in recommended actions, whereas perceived susceptibility is conceptualized as the likelihood that one is subject to the given health threat [18-20]. Additionally, efficacy appraisal is conceptualized as the sum of response efficacy and self-efficacy [18]. Response efficacy refers to the extent to which individuals think recommended protective actions can manage the given threat effectively, whereas self-efficacy is conceptualized as individuals’ confidence in performing those recommended acts [17,18].

The original research on EPPM posits that whether risk perception may facilitate engagement in protective actions depends on the level of efficacy appraisal [17,21]. Specifically, risk perception can only motivate individuals to perform protective actions at high levels of efficacy appraisal, whereas this positive relationship is absent at low levels of efficacy appraisal [17,21]. However, subsequent work demonstrated that risk perception can drive protective actions without high efficacy appraisal [22] because the innate aversion to loss prompts individuals to avoid potential risks by taking preventive measures [23]. Therefore, higher levels of perceived severity and perceived susceptibility may be associated with heightened motivation to perform protective actions [22,24-28].

In addition, individuals reporting high levels of response efficacy are more driven to engage in behaviors that can minimize the threat [26,29,30] because this confidence is often correlated with enhanced levels of hope [31]. Moreover, individuals reporting high levels of self-efficacy are more likely to follow the recommended acts because they tend to think it is less challenging to perform those behaviors [32]. Taken together, we predict that response efficacy and self-efficacy in controlling COVID-19 should exhibit positive relationships with engagement in protective behaviors.

### Information Sources About COVID-19

Individuals equipped with accurate health information are usually more motivated to engage in health behaviors [9]. However, the volume of rumors about COVID-19 makes individuals vulnerable to health misinformation [33]. One factor that could potentially affect the credibility of information is its source. We categorized information sources into expert versus inexpert sources. Expert sources are conceptualized as individuals with medical expertise and organizations with professional gatekeepers that can screen information before it is published. These expert sources include expert media, government administrations, expert health organizations, and medical experts. The gatekeeping theory contends that gatekeepers, or people screening the information in these organizations, can enhance the accuracy of information [34]. Additionally, the heuristic-systematic model suggests that the public is inclined to trust the information provided through these sources because of its authority and thereby more motivated to follow the recommendations that these sources offer [35]. By contrast, inexpert sources are those lacking expertise background or professional gatekeepers, namely celebrities, social media influencers, and social contacts that are not doctors. Therefore, individuals relying on expert versus inexpert sources for information about COVID-19 may demonstrate different patterns of protective behaviors. Given these differences, we predicted that individuals relying on expert sources for information about COVID-19 should be more driven to engage in protective actions whereas reliance on inexpert sources should be related to engagement in protective actions negatively. As mentioned earlier, a repeated cross-sectional investigation will be employed. Hence, an additional question is whether these relationships changed throughout the duration of this study. Three protective actions were assessed: staying at home, wearing a face mask, and washing hands.

## Methods

### Overview

A six-wave repeated cross-sectional survey was conducted between February 7 and April 23, 2020, in collaboration with a large company that provides sampling services in China. Every other week, an online survey was distributed to a convenience sample of residents in six major cities in China. These cities were Beijing, Shanghai, Guangzhou, and Shenzhen, which are the four largest cities in China, as well as Wuhan, where the first COVID-19 cases were discovered [2], and Hangzhou, another city among the cities with the most reported cases [36].

Our survey started on February 7, 2020. Although cases were first found in Wuhan in late December 2019, the Chinese government did not inform the public that COVID-19 could be transmitted between humans until January 20 [37]. On January 23, Wuhan was locked down [38], which started a series of executive orders on travel bans and wearing face mask [13]. We did not start our research until February 7 because January 24 was the Lunar New Year’s Eve, which started a weeklong holiday. Therefore, we could not start our study until early February.

The data collection of wave 1 lasted from February 7-14, 2020. The second wave started on February 20 because most businesses in China restarted by late February and early March [39]. Thus, we wanted to investigate how the resumption of business might have affected our proposed relationships. Given the time difference between these two waves, we decided to collect our data every other week.

The lift of the lockdown in Wuhan on April 7, 2020, signaled the progress of pandemic control [40]. We collected the last wave (April 16-23) of data after April 7 to examine whether the lift of Wuhan’s lockdown might have changed our participants’ responses.

### Sample

Table 1 presents the characteristics of the final sample in each wave. We matched the education and age of our sample to the national population. The most recent national census available to the public shows that around 14% of Chinese people received an associate’s degree or higher [41]. We also used this census to calculate the proportion of age strata in our sample: aged 18-30 years (19%), 31-45 years (26%), and 46 years and older (55%). However, this quota of education and age did not always match our sample characteristics in all waves.

Across all waves, there was no significant difference in biological sex (*χ*^2^_5_=5.56, *P*=.35) and city of residence (*χ*^2^_25_=6.99, *P*>.99). However, our participants differed significantly between waves in their age (*F*_5,901.48_=5.75, *P*<.001; one assumption of one-way variance of analysis is the homogeneity of variances in the dependent variable; however, this assumption was violated when age was compared across waves, so Welch was used to compare differences between waves), education (*χ*^2^_5_=27.49, *P*<.001), and income (*χ*^2^_5_=44.88, *P*<.001).

**Table 1:** Sample characteristics across waves.

Characteristics		Wave 1 (n=321)	Wave 2 (n=319)	Wave 3 (n=315)	Wave 4 (n=343)	Wave 5 (n=329)	Wave 6 (n=315)
**Sex, n (%)**							
	Male	154 (48)	164 (51.4)	141 (44.8)	157 (45.8)	153 (46.5)	163 (51.7)
	Female	167 (52)	155 (48.6)	174 (55.2)	186 (54.2)	176 (53.5)	152 (48.3)
**Age (years), n (%)**							
	18-30	55 (17.1)	64 (20.1)	63 (20)	84 (24.5)	74 (22.5)	60 (19)
	31-45	97 (30.2)	82 (25.7)	87 (27.6)	110 (32.1)	86 (26.1)	80 (25.4)
	≥46	169 (52.6)	173 (54.2)	165 (52.4)	149 (43.4)	169 (51.4)	175 (55.6)
**Education, n (%)**							
	Middle school or lower	38 (11.8)	21 (6.6)	24 (7.6)	10 (2.9)	23 (7)	50 (15.9)
	High school	234 (72.9)	252 (79)	249 (79)	265 (77.3)	250 (76)	222 (70.5)
	Associate’s degree or higher	49 (15.3)	46 (14.4)	42 (13.3)	68 (19.8)	56 (17)	43 (13.7)
**Household monthly income (US $)**							
	≤500, n (%)	15 (4.7)	9 (2.8)	14 (4.4)	7 (2)	14 (4.3)	25 (7.9)
	501-714.29, n (%)	31 (9.7)	29 (9.1)	22 (7)	25 (7.3)	21 (6.4)	36 (11.4)
	714.3-1142.86, n (%)	55 (17.1)	41 (12.9)	48 (15.2)	55 (16)	49 (14.9)	75 (23.8)
	1142.87-1785.71, n (%)	81 (25.2)	95 (29.8)	87 (27.6)	93 (27.1)	93 (28.3)	89 (28.3)
	1785.72-5500, n (%)	113 (35.2)	126 (39.5)	128 (40.6)	138 (40.2)	130 (39.5)	79 (25.1)
	5500.01-11,928.57, n (%)	20 (6.2)	9 (2.8)	9 (2.9)	16 (4.7)	16 (4.9)	8 (2.5)
	≥11,928.58, n (%)	6 (1.9)	10 (0.31)	7 (2.2)	9 (2.6)	6 (1.8)	3 (1)
	Mean (SD)	4.03 (1.32)	4.15 (1.23)	4.1 (1.24)	4.21 (1.19)	4.14 (1.24)	3.63 (1.31)
**City of residence, n (%)**							
	Beijing	55 (17.1)	53 (16.6)	52 (16.5)	59 (17.2)	52 (15.8)	51 (16.2)
	Shanghai	54 (16.8)	53 (16.6)	51 (16.2)	58 (16.9)	52 (15.8)	52 (16.5)
	Guangzhou	53 (16.5)	53 (16.6)	54 (17.1)	71 (20.7)	54 (16.4)	50 (15.9)
	Shenzhen	53 (16.5)	51 (16)	53 (16.8)	51 (14.9)	64 (19.5)	53 (16.8)
	Wuhan	52 (16.2)	53 (16.6)	52 (16.5)	50 (14.6)	53 (16.1)	53 (16.8)
	Hangzhou	54 (16.8)	56 (17.6)	53 (16.6)	54 (15.7)	54 (16.4)	56 (17.8)

### Measures

Table 2 presents the reliability and descriptive statistics of independent and dependent variables in this study. Reliance on expert sources was measured by asking participants to indicate the extent to which their major source of information about COVID-19 was government health departments, government administrations, official media, medical institutes, medical experts, family and friends who are doctors, or the World Health Organization and other health organizations outside China (1=strongly disagree, 7=strongly agree). Reliance on inexpert sources was assessed by the same question except that the sources were replaced with celebrities, social media influencers, family and friends who are not doctors, and other social contacts who are not doctors. The reliability of these two variables at all waves reached .7 or above, except for reliance on inexpert sources, which was .66 at wave 4.

Gore and Bracken’s [42] 7-point Likert scale (1=strongly disagree, 7=strongly agree) was adapted to measure perceived severity, perceived susceptibility, response efficacy, and self-efficacy in controlling COVID-19. Specifically, *perceived severity* was measured with three questions (“COVID-19 is a very serious disease/will pose a severe threat to my health/will pose a severe threat to others’ safety”), and *perceived susceptibility* was measured with two items (“My chance to get COVID-19 is high” and “I can get COVID-19 from others”). *Response efficacy* was assessed with two items (“modern medical knowledge can control COVID-19” and “COVID-19 can be cured as long as one follows doctors’ recommendations”), and *self-efficacy* was assessed with three items (“I can follow the recommended acts to protect myself from COVID-19,” “I have no difficulty in performing those protective behaviors that the government recommended,” “I can master how to perform recommended actions”). The reliability of these three variables reached .7 or above across all waves except for self-efficacy, which was .69 at wave 2.

**Table 2:** Cronbach alpha, means, and SDs of major variables.

Variables	Wave 1		Wave 2		Wave 3		Wave 4		Wave 5		Wave 6	
	α	Mean (SD)	α	Mean (SD)	α	Mean (SD)	α	Mean (SD)	α	Mean (SD)	α	Mean (SD)
Expert sources	.78	5.41 (0.85)	.79	5.61 (0.79)	.73	5.61 (0.68)	.77	5.64 (0.74)	.78	5.60 (0.71)	.78	5.76 (0.73)
Inexpert sources	.70	4.00 (1.04)	.75	3.95 (1.04)	.75	3.84 (1.03)	.66	3.93 (0.92)	.80	3.87 (1.04)	.78	3.98 (1.08)
Perceived severity	.80	6.09 (1.03)	.83	6.26 (0.99)	.78	6.10 (1.00)	.81	6.23 (0.99)	.72	6.08 (0.91)	.81	6.24 (0.98)
Perceived susceptibility	.72	4.24 (1.56)	.73	4.30 (1.63)	.79	3.92 (1.59)	.70	4.05 (1.50)	.74	4.06 (1.42)	.83	4.26 (1.59)
Response efficacy	.70	5.42 (1.18)	.80	5.28 (1.33)	.72	5.39 (1.17)	.77	5.35 (1.27)	.70	5.32 (1.14)	.72	5.57 (1.13)
Self-efficacy	.77	5.89 (0.95)	.69	5.86 (0.90)	.71	5.83 (0.87)	.70	5.95 (0.82)	.73	5.84 (0.81)	.78	5.90 (0.91)
Staying at home	N/A^a^	4.10 (1.00)	N/A	4.15 (0.99)	N/A	3.99 (0.96)	N/A	3.76 (1.08)	N/A	3.60 (1.09)	N/A	3.57 (1.11)
Wearing a face mask	N/A	4.75 (0.89)	N/A	4.78 (0.81)	N/A	4.79 (0.78)	N/A	4.81 (0.74)	N/A	4.80 (0.70)	N/A	4.70 (0.86)
Washing hands	N/A	4.72 (0.81)	N/A	4.80 (0.57)	N/A	4.75 (0.64)	N/A	4.70 (0.75)	N/A	4.74 (0.60)	N/A	4.68 (0.67)

^a^N/A: not applicable.

Personal engagement in protective measures was assessed through three 5-point Likert questions. Participants were asked how often they went out during the past 7 days (1=never, 2=once or twice, 3=three or four times, 4=five or six times, 5=seven times or more). We reverse coded participants’ response to this question, so the large number indicates *staying at home* more often. We also asked participants how often they wore a face mask and washed their hands during the past 7 days (1=never, 2=rarely, 3=sometimes, 4=often, 5=all the time). Again, larger numbers indicate higher frequency of *wearing a face mask* and *washing hands*.

Control variables were biological sex, age, education (recoded as 1=middle school or lower, 2=high school, 3=associate’s degree or higher), household monthly income, city of residence, self-rated health condition (1=very unhealthy, 5=very healthy), and knowledge. *Knowledge* was measured with 17 questions on the transmission of COVID-19, its medication, vulnerable population, and prevention methods. Participants received one point whenever they made a correct option. This made the maximum score 42 points.

### Data Analysis

We employed the Kruskal-Wallis H test to examine if there was any difference between engagement in the three protective behaviors and if the level of engagement in these behaviors differed across time. In addition, we conducted repeated ordinal regression through SPSS 25 (IBM Corp) to test our predictions. At each wave, the dependent variables were entered into the model separately, along with control variables and independent variables. This analysis was repeated six times. Log odds ratios (ORs) and ORs along with their 95% CIs were reported to indicate the relationship between two variables.

The ordinal regression results are shown in the tables in the next section. Given the volume of these findings, results were presented separately with different sets of independent variables. Yet, ordinal regression was conducted with all independent variables listed in the tables.

## Results

### Engagement in Protective Behaviors

Table 3 presents results of the comparisons between engagement in three protective behaviors. Significant differences were found in staying at home across all waves (*χ*^2^_5_=110.01, *P*<.001). However, no significant differences were found in wearing a face mask (*χ*^2^_5_=8.07, *P*=.15) and washing hands (*χ*^2^_5_=10.81, *P*=.06) across time. In addition, across all six waves, we found significant differences consistently in the level of engagement in all three behaviors (Table 3).

**Table 3:** Differences in engagement in three protective behaviors across time.

Behaviors	Wave 1	Wave 2	Wave 3	Wave 4	Wave 5	Wave 6	Chi-square (*df*)	*P* value
Staying at home, mean	4.10	4.15	3.99	3.76	3.60	3.57	110.01 (5)	<.001
Wearing a face mask, mean	4.75	4.78	4.79	4.81	4.80	4.70	8.07 (5)	.15
Washing hands, mean	4.72	4.80	4.75	4.70	4.74	4.68	10.81 (5)	.06
Chi-square (*df*)	205.69 (2)	225.46 (2)	252.35 (2)	331.43 (2)	367.66 (2)	344.63 (2)	N/A^a^	N/A
*P* value	<.001	<.001	<.001	<.001	<.001	<.001	N/A	N/A

^a^N/A: not applicable.

### The Effects of Perceived Severity and Perceived Susceptibility

Table 4 presents how perceived severity and perceived susceptibility predicted engagement in the three protective behaviors across time. Perceived severity of COVID-19 predicted staying at home positively at waves 2 and 6 (Table 4). Individuals perceiving COVID-19 as more severe were more likely to wear a face mask at waves 1 and 5 (Table 4). The effect of perceived severity on washing hands was significant at waves 2, 4, 5, and 6 (Table 4). Conversely, perceived susceptibility to COVID-19 only predicted staying at home at waves 1 and 3, and both relationships were negative (Table 4). The effects of perceived susceptibility on wearing a face mask and washing hands were not significant.

**Table 4:** The effects of perceived severity and perceived susceptibility on engagement in protective behaviors across time.

Time and protective behaviors		Perceived severity		Perceived susceptibility	
		Log OR^a^ (95% CI)	OR (95% CI)	Log OR (95% CI)	OR (95% CI)
**Wave 1**					
	Staying at home	–0.18 (–0.42 to 0.06)	0.84 (0.66 to 1.06)	–0.22 (–0.37 to –0.07)**	0.80 (0.69 to 0.93)
	Wearing a face mask	0.46 (0.05 to 0.87)*	1.59 (1.06 to 2.39)	–0.07 (–0.38 to 0.24)	0.93 (0.69 to 1.27)
	Washing hands	0.31 (–0.04 to 0.66)	1.36 (0.96 to 1.93)	0.01 (–0.24 to 0.26)	1.01 (0.79 to 1.30)
**Wave 2**					
	Staying at home	0.26 (0.02 to 0.51)*	1.30 (1.02 to 1.66)	–0.06 (–0.20 to 0.09)	0.95 (0.82 to 1.09)
	Wearing a face mask	0.34 (–0.09 to 0.77)	1.41 (0.92 to 2.17)	–0.29 (–0.63 to 0.04)	0.75 (0.53 to 1.04)
	Washing hands	0.53 (0.20 to 0.85)**	1.69 (1.22 to 2.35)	–0.07 (–0.31 to 0.17)	0.93 (0.73 to 1.19)
**Wave 3**					
	Staying at home	–0.12 (–0.36 to 0.11)	0.89 (0.70 to 1.12)	–0.21 (–0.35 to –0.06)**	0.81 (0.70 to 0.94)
	Wearing a face mask	0.05 (–0.36 to 0.47)	1.06 (0.70 to 1.60)	–0.15 (–0.45 to 0.14)	0.86 (0.64 to 1.16)
	Washing hands	–0.06 (–0.40 to 0.27)	0.94 (0.67 to 1.31)	–0.16 (–0.37 to 0.05)	0.85 (0.69 to 1.05)
**Wave 4**					
	Staying at home	–0.16 (–0.38 to 0.06)	0.85 (0.68 to 1.06)	–0.06 (–0.20 to 0.09)	0.94 (0.82 to 1.09)
	Wearing a face mask	0.04 (–0.37 to 0.45)	1.04 (0.69 to 1.57)	–0.02 (–0.34 to 0.31)	0.99 (0.72 to 1.36)
	Washing hands	0.37 (0.10 to 0.64)**	1.45 (1.10 to 1.90)	–0.15 (–0.38 to 0.07)	0.86 (0.69 to 1.07)
**Wave 5**					
	Staying at home	0.11 (–0.14 to 0.36)	1.12 (0.87 to 1.43)	0.01 (–0.15 to 0.16)	1.01 (0.86 to 1.18)
	Wearing a face mask	0.55 (0.12 to 0.98)*	1.73 (1.13 to 2.66)	0.12 (–0.19 to 0.43]	1.13 (0.83 to 1.54)
	Washing hands	0.41 (0.07 to 0.74)*	1.50 (1.07 to 2.10)	–0.08 (–0.31 to 0.15)	0.92 (0.73 to 1.16)
**Wave 6**					
	Staying at home	0.28 (0.04 to 0.51)*	1.32 (1.04 to 1.66)	0.002 (–0.14 to 0.14)	1.00 (0.87 to 1.15)
	Wearing a face mask	0.26 (–0.05 to 0.57)	1.30 (0.95 to 1.77)	–0.06 (–0.31 to 0.18)	0.94 (0.74 to 1.20)
	Washing hands	0.31 (0.03 to 0.59)*	1.36 (1.03 to 1.81)	–0.09 (–0.40 to 0.23)	0.92 (0.67 to 1.25)

^a^OR: odds ratio.

*P<.05.

**P<.01.

### The Effects of Response Efficacy and Self-Efficacy

Table 5 shows how response efficacy and self-efficacy affected engagement in protective actions across time. At wave 1, response efficacy predicted staying at home and washing hands positively (Table 5). After wave 1, its effect on protective behaviors became weak. Individuals who reported higher levels of response efficacy were more likely to stay at home at wave 2 and wash hands at wave 4 (Table 5). Response efficacy was not significantly associated with wearing a face mask at any time. Self-efficacy did not predict any protective behavior at any time.

**Table 5:** The effects of response efficacy and self-efficacy on engagement in protective behaviors across time.

Time and protective behaviors		Response efficacy		Self-efficacy	
		Log OR^a^ (95% CI)	OR (95% CI)	Log OR (95% CI)	OR (95% CI)
**Wave 1**					
	Staying at home	0.30 (0.08 to 0.52)**	1.35 (1.08 to 1.68)	–0.04 (–0.33 to 0.26)	0.97 (0.72 to 1.30)
	Wearing a face mask	–0.01 (–0.42 to 0.41)	0.93 (0.69 to 1.27)	0.24 (–0.27 to 0.74)	1.27 (0.77 to 2.10)
	Washing hands	0.36 (0.04 to 0.68)*	1.43 (1.04 to 1.97)	–0.10 (–0.53 to 0.34)	0.91 (0.59 to 1.40)
**Wave 2**					
	Staying at home	0.31 (0.11 to 0.50)**	1.36 (1.11 to 1.66)	–0.04 (–0.34 to 0.27)	0.96 (0.71 to 1.31)
	Wearing a face mask	0.05 (–0.36 to 0.46)	1.05 (0.70 to 1.59)	0.24 (–0.33 to 0.81)	1.27 (0.73 to 2.24)
	Washing hands	0.01 (–0.30 to 0.32)	1.01 (0.74 to 1.38)	0.37 (–0.10 to 0.83)	1.45 (0.91 to 2.30)
**Wave 3**					
	Staying at home	0.08 (–0.13 to 0.28)	1.08 (0.88 to 1.32)	–0.15 (–0.44 to 0.14)	0.86 (0.64 to 1.15)
	Wearing a face mask	–0.07 (–0.48 to 0.35)	0.94 (0.62 to 1.42)	–0.21 (–0.76 to 0.34)	0.81 (0.47 to 1.40)
	Washing hands	–0.14 (–0.43 to 0.15)	0.87 (0.65 to 1.17)	0.25 (–0.15 to 0.65)	1.28 (0.86 to 1.91)
**Wave 4**					
	Staying at home	–0.09 (–0.27 to 0.08)	0.91 (0.77 to 1.09)	0.02 (–0.28 to 0.31)	1.02 (0.76 to 1.37)
	Wearing a face mask	0.25 (–0.09 to 0.58)	1.28 (0.92 to 1.79)	0.03 (–0.52 to 0.58)	1.04 (0.60 to 1.79)
	Washing hands	0.26 (0.01 to 0.51)*	1.30 (1.01 to 1.66)	–0.07 (–0.47 to 0.33)	0.94 (0.63 to 1.39)
**Wave 5**					
	Staying at home	0.18 (–0.04 to 0.39)	1.19 (0.96 to 1.48)	–0.17 (–0.47 to 0.13)	0.84 (0.63 to 1.14)
	Wearing a face mask	0.15 (–0.27 to 0.58)	1.17 (0.76 to 1.78)	–0.15 (–0.70 to 0.40)	0.86 (0.50 to 1.49)
	Washing hands	0.02 (–0.29 to 0.33)	1.02 (0.75 to 1.39)	0.28 (–0.13 to 0.69)	1.33 (0.88 to 2.00)
**Wave 6**					
	Staying at home	0.03 (–0.20 to 0.25)	1.03 (0.82 to 1.28)	0.05 (–0.26 to 0.36)	1.05 (0.77 to 1.43)
	Wearing a face mask	–0.13 (–0.52 to 0.27)	0.88 (0.59 to 1.31)	0.15 (–0.32 to 0.62)	1.16 (0.73 to 1.86)
	Washing hands	–0.09 (–0.40 to 0.23)	0.92 (0.67 to 1.25)	0.19 (–0.19 to 0.58)	1.21 (0.83 to 1.78)

^a^OR: odds ratio.

*P<.05.

**P<.01.

### The Effects of Reliance on Expert Versus Inexpert Sources

Table 6 demonstrates how individuals’ reliance on expert versus inexpert sources for information about COVID-19 might affect their engagement in the three protective actions across time. Reliance on expert sources did not predict engagement in any protective behaviors at wave 1, and only predicted wearing a face mask at wave 2 (Table 6). Starting from wave 3, the facilitating effect of expert sources became more prominent. Specifically, reliance on expert sources predicted staying at home positively at waves 3 and 4 (Table 6). In addition to wave 2, individuals relying on expert sources for information about COVID-19 were more likely to wear a face mask at waves 4, 5, and 6 (Table 6). The relationship between reliance on expert sources and washing hands was significant at waves 3, 4, 5, and 6 (Table 6).

The effect of reliance on inexpert sources on protective behaviors was more limited. Reliance on inexpert sources exhibited a *negative* effect on *staying at home* at wave 2 (Table 6). Individuals relying on inexpert sources were *less* likely to *wear a face mask* at wave 2 and *wash hands* at wave 5 (Table 6).

**Table 6:** The effects of reliance on expert versus inexpert sources on engagement in protective behaviors across time.

Time and protective behaviors		Expert sources		Inexpert sources	
		Log OR^a^ (95% CI)	OR (95% CI)	Log OR (95% CI)	OR (95% CI)
**Wave 1**					
	Staying at home	0.06 (–0.25 to 0.37)	1.06 (0.78 to 1.45)	0.04 (–0.18 to 0.25)	1.04 (0.84 to 1.28)
	Wearing a face mask	–0.06 (–0.71 to 0.60)	0.94 (0.49 to 1.81)	–0.22 (–0.66 to 0.22)	0.80 (0.52 to 1.24)
	Washing hands	0.33 (–0.16 to 0.82)	1.39 (0.85 to 2.28)	–0.34 (–0.72 to 0.05)	0.71 (0.49 to 1.05)
**Wave 2**					
	Staying at home	0.34 (–0.01 to 0.69)	1.40 (0.99 to 1.99)	–0.25 (–0.49 to –0.02)*	0.78 (0.62 to 0.99)
	Wearing a face mask	0.68 (0.01 to 1.34)*	1.97 (1.01 to 3.83)	–0.50 (–0.97 to –0.03)*	0.61 (0.38 to 0.97)
	Washing hands	0.39 (–0.13 to 0.92)	1.48 (0.87 to 2.51)	–0.27 (–0.65 to 0.12)	0.77 (0.52 to 1.12)
**Wave 3**					
	Staying at home	0.50 (0.11 to 0.89)*	1.64 (1.11 to 2.42)	–0.20 (–0.43 to 0.03)	0.83 (0.65 to 1.03)
	Wearing a face mask	0.64 (–0.12 to 1.40)	1.89 (0.89 to 4.05)	–0.06 (–0.48 to 0.36)	0.94 (0.62 to 1.43)
	Washing hands	0.76 (0.21 to 1.30)**	2.13 (1.23 to 3.68)	–0.15 (–0.48 to 0.17)	0.86 (0.62 to 1.19)
**Wave 4**					
	Staying at home	0.49 (0.17 to 0.82)**	1.64 (1.18 to 2.27)	–0.10 (–0.34 to 0.14)	0.91 (0.71 to 1.15)
	Wearing a face mask	0.82 (0.20 to 1.44)*	2.26 (1.22 to 4.22)	–0.34 (–0.89 to 0.20)	0.71 (0.41 to 1.23)
	Washing hands	0.96 (0.50 to 1.42)***	2.61 (1.65 to 4.12)	–0.04 (–0.39 to 0.31)	0.96 (0.68 to 1.36)
**Wave 5**					
	Staying at home	0.01 (–0.34 to 0.35)	1.01 (0.71 to 1.42)	–0.08 (–0.30 to 0.14)	0.92 (0.74 to 1.15)
	Wearing a face mask	0.64 (0.09 to 1.20)*	1.90 (1.09 to 3.30)	–0.35 (–0.80 to 0.11)	0.71 (0.45 to 1.11)
	Washing hands	0.59 (0.13 to 1.05)*	1.80 (1.14 to 2.85)	–0.37 (–0.71 to –0.03)*	0.69 (0.49 to 0.97)
**Wave 6**					
	Staying at home	0.08 (–0.30 to 0.46)	1.08 (0.74 to 1.58)	–0.22 (–0.44 to 0.003)	0.80 (0.64 to 1.00)
	Wearing a face mask	0.64 (0.09 to 1.19)*	1.90 (1.10 to 3.28)	–0.19 (–0.54 to 0.16)	0.83 (0.58 to 1.17)
	Washing hands	0.74 (0.27 to 1.22)**	2.10 (1.31 to 3.39)	–0.05 (–0.34 to 0.24)	0.95 (0.71 to 1.28)

^a^OR: odds ratio.

*P<.05.

**P<.01.

***P<.001.

### The Effects of Control Variables

Knowledge did not predict *staying at home* at any time. Individuals equipped with more knowledge were more likely to *wear a face mask* at wave 3 (OR 1.14, 95% CI 1.05-1.25; *P*<.01). The relationship between knowledge and *washing hands* was only significant and positive at wave 3 (OR 1.09, 95% CI 1.02-1.17; *P*<.05) and wave 6 (OR 1.08, 95% CI 1.01-1.15; *P*<.05).

The self-rated health condition predicted *wearing a face mask* (OR 2.21, 95% CI 1.27-3.85; *P*<.01) and *washing hands* positively at wave 4 (OR 1.63, 95% CI 1.10-2.41; *P*<.05). At wave 6, the relationship between self-rated health condition and *staying at home* was positive (OR 1.43, 95% CI 1.02-2.00; *P*<.05).

Income predicted *staying at home negatively* at wave 3 (OR 0.77, 95% CI 0.64-0.93; *P*<.01), wave 4 (OR 0.81, 95% CI 0.67-0.97; *P*<.05), and wave 6 (OR 0.83, 95% CI 0.69-1.00; *P*<.01). Individuals with a greater household monthly income were *more* likely to *wear a face mask* at wave 1 (OR 1.67, 95% CI 1.19-2.35; *P*<.01), wave 2 (OR 1.49, 95% CI 1.02-2.17; *P*<.05), wave 4 (OR 1.47, 95% CI 1.00-2.16; *P*<.05), and wave 6 (OR 1.43, 95% CI 1.08-1.89; *P*<.05). The relationship between income and *washing hands* was *positive* at wave 1 (OR 1.37, 95% CI 1.04-1.79; *P*<.05) and wave 4 (OR 1.37, 95% CI 1.06-1.77; *P*<.05).

Compared to women, men *washed hands* less often at wave 4 (OR 0.38, 95% CI 0.20-0.72; *P*<.01), wave 5 (OR 0.43, 95% CI 0.23-0.80; *P*<.01), and wave 6 (OR 0.50, 95% CI 0.28-0.92; *P*<.05). *Age* predicted *washing hands* positively at wave 1 (OR 1.04, 95% CI 1.01-1.08; *P*<.05), wave 4 (OR 1.06, 95% CI 1.02-1.09; *P*<.01), and wave 6 (OR 1.04, 95% CI 1.00-1.07; *P*<.05). At wave 4, participants with a high school degree *washed hands* less often than those with an associate’s degree or above (OR 0.36, 95% CI 0.15-0.89; *P*<.05).

When it comes to city differences, residents in Wuhan, where COVID-19 cases were first discovered, were used as the reference group. No significant difference was found in *wearing a face mask* and *washing hands* across all waves, except that residents in Beijing reported to *wear a face mask* more often than those in Wuhan (OR 9.69, 95% CI 1.09-86.38; *P*<.05). However, residents in Wuhan *stayed at home* more often than those in the other cities at most times, as Tables 7 and 8 shows.

**Table 7:** City differences in staying at home waves 1, 2, and 3 (Wuhan was used as the reference group).

City	Wave 1		Wave 2		Wave 3	
	Log OR^a^ (95% CI)	OR (95% CI)	Log OR (95% CI)	OR (95% CI)	Log OR (95% CI)	OR (95% CI)
Beijing	–0.62 (–1.38 to 0.13)	0.54 (0.25 to 1.14)	–1.76 (–2.57 to –0.95)***	0.17 (0.08 to 0.39)	–0.84 (–1.60 to –0.07)*	0.43 (0.20 to 0.93)
Shanghai	–0.99 (–1.75 to –0.22)*	0.37 (0.17 to 0.80)	–1.74 (–2.56 to –0.92)***	0.18 (0.08 to 0.40)	–1.32 (–2.10 to –0.54)**	0.27 (0.12 to 0.58)
Guangzhou	–0.87 (–1.62 to –0.11)*	0.42 (0.20 to 0.89)	–1.75 (–2.58 to –0.92)***	0.17 (0.08 to 0.40)	–0.72 (–1.49 to 0.04)	0.49 (0.23 to 1.04)
Shenzhen	–01.15 (–1.93 to –0.36)**	0.32 (0.15 to 0.70)	–1.58 (–2.41 to –0.75)***	0.21 (0.09 to 0.47)	–0.81 (–1.58 to –0.04)*	0.44 (0.21 to 0.96)
Hangzhou	–1.01 (–1.77 to –0.25)**	0.37 (0.17 to 0.78)	–1.33 (–2.18 to 0.47)**	0.27 (0.11 to 0.62)	–1.18 (–1.95 to –0.42)**	0.31 (0.14 to 0.66)

^a^OR: odds ratio.

*P<.05.

**P<.01.

***P<.001.

**Table 8:** City differences in staying at home waves 4, 5, and 6 (Wuhan was used as the reference group).

City	Wave 4		Wave 5		Wave 6	
	Log OR^a^ (95% CI)	OR (95% CI)	Log OR (95% CI)	OR (95% CI)	Log OR (95% CI)	OR (95% CI)
						
Beijing	–1.26 (–2.02 to –0.51)**	0.28 (0.13 to 0.60)	–0.14 (–0.90 to 0.61)	0.87 (0.41 to 1.84)	–0.49 (–1.26 to 0.27)	0.61 (0.28 to 1.31)
Shanghai	–1.35 (–2.11 to –0.58)**	0.26 (0.12 to 0.56)	–0.63 (–1.40 to 0.14)	0.53 (0.25 to 1.15)	–0.99 (–1.78 to –0.21)*	0.37 (0.17 to 0.81)
Guangzhou	–1.35 (–2.07 to –0.64)***	0.26 (0.13 to 0.53)	–1.28 (–2.04 to –0.52)**	0.28 (0.13 to 0.59)	–0.52 (–1.32 to 0.28)	0.59 (0.27 to 1.32)
Shenzhen	–1.25 (–2.02 to –0.48)**	0.29 (0.13 to 0.62)	–0.73 (–1.44 to –0.02)*	0.48 (0.24 to 0.99)	–0.44 (–1.23 to 0.35)	0.65 (0.29 to 1.42)
Hangzhou	–1.75 (–2.52 to –0.98)***	0.17 (0.08 to 0.38)	–1.02 (–1.75 to –0.30)**	0.36 (0.17 to 0.74)	–0.55 (–1.31 to 0.21)	0.58 (0.27 to 1.23)

^a^OR: odds ratio.

*P<.05.

**P<.01.

***P<.001.

### Summary

Despite inconsistencies, some patterns still emerged. First, reliance on expert sources encouraged protective behaviors, but this effect did not emerge until wave 3 and was stronger on wearing a face mask and washing hands. Second, the discouraging effect of reliance on inexpert sources was limited to wave 2 except that it predicted washing hands negatively at wave 5. In addition, perceived severity exhibited a stronger effect on protective behaviors than perceived susceptibility. Furthermore, self-efficacy was not associated with engaging in protective behaviors, whereas the effect of response efficacy was limited to waves 1 and 2. Among all control variables, the effect of knowledge was limited, whereas the city of residence exhibited a stronger effect on staying at home.

## Discussion

### Principal Findings

The COVID-19 pandemic triggered research on what factors affected individuals’ engagement in protective behaviors [7-12]. This study is built upon EPPM, a theoretical framework that explains how risk perception and efficacy appraisal might affect individuals’ engagement in protective behaviors [13]. In addition, given the volume of misinformation about preventive measures against COVID-19 [33], we extended EPPM and the extant research on protective actions against COVID-19 by recognizing the value of accurate information and considering Chinese individuals’ reliance on expert versus inexpert information sources. Further, differences across time and between three target behaviors were also revealed. The patterns of our findings previously summarized provide important implications on health education and suggest the intertwined relationship between one’s health behavior and the sociocultural system where these individuals reside.

First, we found that perceived severity could encourage protective behaviors, but their effects were not consistent and different depending on the specific behavior. Taken as a whole, perceived severity predicted washing hands positively at waves 2, 4, 5, and 6, more consistently than wearing a face mask (waves 1 and 5) and staying at home (waves 2 and 6). The inconsistency might be related to the executive orders that the Chinese government issued, which forced individuals to wear a face mask in public and placed them in quarantine [13]. Therefore, in this study, wearing a face mask and staying at home were not entirely autonomous decisions but more because of compliance with the executive orders. However, washing hands was not required, and it was impossible to ensure that everyone washed their hands as recommended. Thus, how often individuals washed their hands was likely derived from their evaluation of the risk.

Surprisingly, perceived susceptibility predicted staying at home negatively at waves 1 and 3. The post hoc analysis found that at both waves the common predictor of perceived susceptibility was self-rated health condition (wave 1: OR 0.72, 95% CI 0.56-0.92; *P*<.05; wave 3: OR 0.65, 95% CI 0.50-0.86; *P*<.01), and older participants reported a worse health condition (wave 1: OR 0.97, 95% CI 0.95-0.99; *P*<.01; wave 3: OR 0.95, 95% CI 0.93-0.97; *P*<.001). Therefore, among older participants, there might be a gap between risk perception and behavior. Although they realized that they could be subject to COVID-19, they still went out. This suggests that health education for seniors should focus on bridging the perception-behavior gap.

Overall, the effect of perceived susceptibility on protective behaviors was minimal. However, the impact of perceived susceptibility should not be dismissed. For example, protection motivation theory contends that human behavior is a function of the perceived severity of the threat, perceived susceptibility to the threat, and response efficacy, and no behavior is performed if any of these predictors are zero [14]. Although more empirical evidence is needed to understand whether health education in China during the pandemic lacks information on susceptibility, this result suggested that subsequent education should highlight the chance that certain populations are vulnerable to the pandemic.

In addition to risk perceptions, our results showed that response efficacy only predicted staying at home at waves 1 and 2, and washing hands at wave 1. Hence, at the early stage of the outbreak, individuals engaged in preventive measures because perhaps they believed these actions were effective to protect them against the given threat. This suggests that practitioners may want to adjust the emphasis of health education as time passes. Specifically, elevating response efficacy of the target audience may be important at the early stage of the outbreak.

By contrast, self-efficacy did not predict any protective behavior at any time. One possible reason is that our measure of self-efficacy addressed overall confidence in performing preventive measures instead of specific preventive actions. However, there might be differences in the level of difficulty in performing these three protective behaviors. Thus, our measure might not have assessed this subtle difference.

It is important to note that EPPM research tends to test the aggregate effects of perceived severity and perceived susceptibility as well as response efficacy and self-efficacy on protective behaviors [18,21,22,42,43]. However, we demonstrated the separate effects of these variables, and we found their distinct effects. This suggests that perceived severity versus perceived susceptibility (response efficacy vs self-efficacy) may be essentially different, which needs further study.

In addition to testing EPPM, our results demonstrated how reliance on expert versus inexpert sources might affect Chinese individuals’ engagement in protective actions. Our findings reveal that the positive effect of expert sources did not emerge until wave 3 when most businesses restarted [39]. The post hoc analysis found that, controlling for knowledge, self-rated health condition, and demographic variables, reliance on expert sources at wave 1 was significantly lower than all other waves (wave 2: OR 2.26, 95% CI 1.72-2.98; *P*<.001; wave 3: OR 1.56, 95% CI 1.19-2.04; *P*<.01; wave 4: OR 1.75, 95% CI 1.34-2.29; *P*<.001; wave 5: OR 1.54, 95% CI 1.17-2.02; *P*<.01; wave 6: OR 1.57, 95% CI 1.20-2.06; *P*<.01). One explanation is that it took time for the Chinese public to develop trust in these expert sources and follow the messages that these sources delivered. Expert sources in China, such as official media and health departments, are under strict control by the Chinese government, which was blamed for their failure to provide timely responses to COVID-19 during its early outbreak. This might have affected Chinese individuals’ trust in these expert sources given their close connection with the government. However, the aggressive actions that the government took controlled the spread of the pandemic and made the number of cases start to decline in late February 2020 [3,13]. Therefore, at wave 3, which started in early March, Chinese individuals might have gained more trust in these expert sources, making them more willing to comply with the recommendations that these sources offered. This suggests that individuals’ trust in information sources may exhibit a critical impact on their health behavior. Furthermore, this finding suggests that the conventional approach to persuading the public to engage in protective behaviors during the pandemic, which centers on knowledge provision, may not be effective. A more important mission might be to help the public develop trust in the community of public health practitioners including those working for the government. Therefore, a perspective of public relations is needed in future research and practices on health education.

In contrast, reliance on inexpert sources did not affect protective behaviors most of the time, except that these sources discouraged preventive measures at wave 2. This shows that our participants might have realized the risks of inexpert sources in information provision, so they did not follow this information. Although these findings are promising, information literacy should still be a focus of future health education and campaigns, especially those vulnerable to health misinformation, such as seniors and less educated individuals.

Additionally, the significant effect of reliance on inexpert sources was limited to wave 2. One possible explanation is that the public interest changed as time passed. In January and February 2020, the public may have been concerned about how to control and treat COVID-19. However, the restart of businesses might have signaled that the pandemic was under control. By then, individuals may have been more concerned about economic recession and recovery. Hence, after wave 2, the focus of the information exchanged between inexpert sources might have changed, which made reliance on these sources not significantly related to taking preventive measures.

Finally, the effects of several control variables warrant discussion. The impact of knowledge on protective behaviors was limited, and residents in Wuhan stayed at home more than participants in other cities at most times. These two findings can be explained by the influence of executive orders. The lockdown of Wuhan lasted more than 2 months, so naturally, participants from Wuhan stayed at home more. Additionally, the limited influence of knowledge suggests that Chinese individuals’ engagement in protective behaviors might not be a result of their autonomous decisions but compliance with executive orders. Although this approach to behavior change controlled the spread of COVID-19 in China [3,13], the duration of its effect is questionable, which future research needs to investigate.

### Limitations and Future Research

These findings must be interpreted with several caveats. First, the cross-sectional nature of this study makes it impossible to build causal relationships between variables. Second, our study uses self-reported data. This method relies on participants’ memory and can be subject to social desirability.

In addition, as previously explained, Chinese individuals performed these protective actions partly because of their compliance with strict law enforcement and executive orders issued by China’s government. This might explain why our participants’ responses to questions measuring their engagement in protective behaviors were skewed. Furthermore, this might affect the validity of responses that our participants provided. Hence, social desirability must be considered when results are interpreted.

Although we matched the age and the education level of our sample to the national population in China, the generalizability of our sample may still be a limitation. Moreover, the proportions of education and age did not match the national population at all waves. The significant differences in education, income, and age between waves might have introduced additional variances and affected the validity of our results.

This study was conducted in China during the COVID-19 pandemic. This particular timing and geographic location might limit the generalizability of our results. Cross-cultural comparisons and longitudinal observations can be valuable directions for future research.

Our measures of self-efficacy and knowledge could also affect the validity of our findings. As mentioned earlier, the measure of self-efficacy did not specify the preventive behavior. Moreover, we self-created our scale of knowledge based on relevant information from the media. Established measures based on a manual provided by health departments would be more valid.

It is important to note that our definition of risk perception was limited to cognitive appraisal, which may dismiss the effect of affective responses. Future inquiries are needed to understand how cognitive and affective appraisals of risks may affect individuals’ engagement in protective behaviors during the pandemic.

Finally, as argued earlier, whether Chinese individuals engaged in protective behavior might partly be a result of strict executive orders. Thus, Chinese individuals’ attitude toward the political system may play a part in their engagement in protective behaviors against COVID-19. This implication may also apply in other countries such as the United States, where pandemic control has been politicalized [44,45]. Therefore, future research may need to examine how variables such as political interest and political orientation may affect one’s health behavior.

### Conclusion

This study provides empirical evidence on what affected Chinese individuals’ engagement in protective behaviors against COVID-19 between February and April 2020. Given the authoritarian political system in the media, Chinese individuals’ engagement in protective behavior might not be an entirely autonomous decision but a result of compliance with executive orders. Our findings demonstrate that expert sources did not encourage protective behaviors until the early stage passed, suggesting that it might take time to develop trust in expert sources. Therefore, the effect of health education may depend on information as well as the relationship between practitioners and the public. This suggests that a perspective of public relations should be considered in future research. In addition, perceived severity could motivate some protective measures, but its effect differed depending on the specific behavior. Furthermore, the facilitating effect of perceived severity lasted throughout the duration of the pandemic but that of response efficacy was limited to the early stage. Hence, practitioners may want to adjust the emphasis of health campaigns depending on the stage of the pandemic.

## Abbreviations

EPPM: extended parallel process model
OR: odds ratio
